# The Impact of the First Wave of the Covid-19 Pandemic on Chaplaincy in Health Care: Introduction to an International Survey

**DOI:** 10.1177/1542305021992044

**Published:** 2021-03-17

**Authors:** Anne Vandenhoeck

**Affiliations:** KU Leuven, Leuven, Belgium

## Background of the International Survey

The SARS-CoV-2 syndrome first appeared in Asia in late 2019. By March 2020, the first wave of the Covid-19 pandemic spread rapidly affecting populations globally soon challenging health care providers and facilities in North America and Europe. Initially, distant countries such as Australia had limited Covid-19 case; the height of the first wave hit a few months later. Health care Chaplains, like all health care workers, were affected by this global health crisis challenging the spiritual care they delivered. Joost Verhoef, coordinator of ERICH (European Research Institute for Chaplains in HealthCare), conceived the idea of conducting a survey to learn how the pandemic was challenging spiritual care in the European health care arena. ERICH was receiving all kinds of signals from chaplains that the pandemic was having a major effect on them personally as well their professional practice. Together with Austyn Snowden, senior researcher of ERICH, an online survey for chaplains was developed in an effort to understand how chaplains delivered spiritual care during the first wave of the Covid-19 pandemic. The ERICH group also wanted to identify key changes to usual practice and to examine their impact on chaplains, patients, staff and culture. The ERICH group soon identified interested international partners and broadcasted the survey among their networks and joined the effort to understand the findings. The Chaplaincy Innovation Lab, led by Trace Haythorn, Wendy Cadge and Cate Michelle Desjardins, became the USA-partner, and the Spiritual Health Association, led by Cheryl Holmes and Heather Tan, became the Australian partner. The joint research study (G-2020-1964-R2) was approved by the Ethics Review Board of the KU Leuven in Belgium.

The survey appeared online by the end of May 2020 and remained open for three weeks. The results of this international cooperation and the insights of 1657 chaplain participants worldwide is now available in this special issue of the *Journal of Pastoral Care and Counseling*. The consortium of researchers thanks all participating chaplains for the richness of their responses comprised in 44,000 words of qualitative data. Of note, when data refers to “spiritual care practitioner”, this reference applies to the Australian context as the local name for chaplains or spiritual caregivers. The survey distinguishes between spiritual care conducted by chaplains as spiritual care specialists and spiritual care provided by other health care professionals, i.e. generalist spiritual care. One study limitation is that we could not reach or involve chaplains in Africa or parts of Asia.

## The Survey Results

Cumulatively, the survey reveals how chaplains and their provision of spiritual care fared during the first months of 2020. During this timeframe, health care facilities worldwide were, by and large, sufficiently ill-prepared to manage the demands of the Covid-19 pandemic. Shortages of material (protective gear, masks, ventilators, …), limited precise knowledge about the nature of virus, and a general lack of common practice guidelines regarding the involvement of health care professionals at the bedside for physicians, nurses and other care providers. Most noticeably, the survey reveals the differences among chaplains with regard to the care for patients/residents and family members (both Covid and non-Covid patients or residents). Some chaplains were fired or sent home because their management questioned the importance of their professional contribution during this crisis or that they might constitute a risk factor in containing the virus within the health care facility. On the other end of the extreme, some chaplains who received more assignments and recognition, were involved in policy making, and became more integrated and present in their institutions. The majority of chaplains reported being situated somewhere in between these two poles.

The survey provided some insights in to the following questions: What caused the differences? What was the difference between those who are made redundant and those who came out more integrated? The results revealed many factors that contributed to these differences, some of which are included in a number of articles in this volume.

The lead article in this special issue reports the quantitative data. Austyn Snowden, notes that the majority of chaplains in three continents felt that they could have been better deployed during the first wave. The circumstances (for example lack of protective gear) prohibited them from participating fully in the care for patients/residents but other findings also point to the confusion around the role of the chaplain in health care. Chaplains themselves were unclear about their roles and responsibilities as were others.

Anne Vandenhoeck, Cheryl Palmer, Cate Michelle Desjardins and Joost Verhoef reflect on what chaplains reported they lost in spiritual care during the first wave of the pandemic. The loss of physical touch and presence with patients/residents weighs heavily. Not being able to give care while knowing people are in need, especially at the end of life, was a burden. Chaplains also reported a change in their spiritual care due to the massive use of new technologies, something they consider a gain.

Staff care became important as direct presence with patients/residents proved to be difficult. Wendy Cadge, Leila Karimi, Daniel Nuzum, Karen Murphy and Beba Tata reflect on how chaplaincy was valued as a resource for staff support. This finding suggests that providing such care can be an important pathway by which to inform staff’s understanding of the chaplain’s role and of what spiritual care can mean in the context of wholistic care.

Although there were no specific questions in the survey on self-care of chaplains, the authors of the next contribution regarded some responses to this issue embedded in the data. Cate Michelle Desjardins, Martijn Steegen, Mario Cagna, Anna Bovo and Anne Vandenhoeck discerned a mix of strong positive and negative emotions in the answers. In self-care chaplains are challenged to deal with a range of emotions due to the circumstances in care they find themselves in.

The first wave of the pandemic hit health care facilities in Australia in the summer of 2020, later than its impact in Europe and the USA. The responses of Australian spiritual care practitioners were therefore discussed in a different contribution. Heather Tan, Cheryl Holmes, Eleanor Flynn, and Leila Karimi provided a case study of the situation of spiritual care during Covid-19 in Australia.

Chaplains reported changes in spiritual care due to the impact of the pandemic on health care suggest expanded areas for professional research, education, and development. Eleanor Flynn, Heather Tan and Anne Vandenhoeck reflect on the consequences of the findings for educational programs and training for chaplains.

Spiritual care in mental health institutions raised its own set of professional concerns. Some patients and staff became infected but the treatment of all patients, including their spiritual care, became different. Groups sessions or activities, for example, were no longer taking place and visits were no longer permitted. Angelika Zollfrank, John Swinton and David Glenister reflect on the effects of the pandemic on spiritual care in mental health hospitals and institutions.

Megan Best, Geila Rajaee and Anne Vandenhoeck discuss in their contribution the moral injuries reported by chaplains in the survey, the confusion about their contribution, the difference between generalist and specialist spiritual care as well as the strength and weaknesses of the survey.

Looking at the whole of the survey and the different contributions of authors, Trace Haythorn looks towards the future of chaplaincy emphasizing some important findings and considering them within the frame of professional chaplaincy in a multicultural and challenged international scene.

In January 2021 in most parts of the world we see no end yet to the burden on health care systems and health care professionals caused by the pandemic. Chaplains and all other health care professionals are getting tired of the continuous strain on their capacities to deliver quality of care. The pandemic continues to challenge spiritual care and chaplains. The survey reveals much about how spiritual care was effected on a global level. It also reveals how adaptive, creative, and resilient chaplains can be. The team of authors contributing to this special issue thank and honor all chaplains for their continuous care for patients/residents, families and staff, especially those chaplains who became infected themselves and those that did not survive.

